# Molecular detection of *Dientamoeba fragilis* in children in southern Xinjiang, China

**DOI:** 10.1016/j.fawpar.2025.e00275

**Published:** 2025-07-04

**Authors:** Yafei Zhao, Wenxuan Ma, Duoduo Su, Zhenjie Zhang, Aiyun Zhao, Fuchang Yu, Meng Qi

**Affiliations:** a College of Animal Science and Technology, Tarim University, Alaer 843300, Xinjiang, China; b Engineering Laboratory of Tarim Animal Diseases Diagnosis and Control, Xinjiang Production & Construction Corps, China; c Key Laboratory of Livestock and Forage Resources Utilization around Tarim, Ministry of Agriculture and Rural Affairs, China

**Keywords:** *Dientamoeba fragilis*, Detection, PCR, Genotypes

## Abstract

*Dientamoeba fragilis* is a common intestinal parasite in human and animals worldwide. In this study, 609 fecal samples were collected from preschool children in 11 counties in Southern Xinjiang, China. All samples were screened for *D. fragilis* using PCR targeting *SSU* rRNA gene, revealing an infection rate of 4.4 % (27/609). Seven of the 11 counties were *D. fragilis*-positive. The highest infection rate was 15.9 % (10/63) in Yopurga, and the lowest infection rate was 0.9 % (1/109) in Lop. Infection rates in boys and girls were 4.4 % (13/299) and 4.5 % (14/310), respectively. Genetic analysis identified all 27 positive samples as genotype 1. These results confirmed the presence of *D. fragilis* in children in southern Xinjiang, China. The high degree of sequence homology in the *SSU* rRNA gene indicates a clonal distribution pattern for *D. fragilis*.

## Highlights

• *D. fragilis* infection rate in Xinjiang children: 4.4 % (27/609), highest in Yopurga (15.9 %).
• All 27 *D. fragilis* isolates were identified as genotype 1 at the *SSU* rRNA gene.
• No gender difference (boys 4.4 %, girls 4.5 %); significant regional variation (*p* = 0.001).
• Possibility of zoonotic transmission of *D. fragilis* in China should not be ignored.

## 1 Introduction

*Dientamoeba fragilis* is a unicellular protozoan parasite that infects both humans and animals, including livestock, wildlife, and companion animal worldwide (Cacciò, 2018). The pathogenicity of *D. fragilis* in humans is a highly debated topic in the literature (Malatyali et al., 2024). While infected hosts may be asymptomatic, clinical symptoms including altered bowel motility, intermittent diarrhea, anal pruritus, abdominal pain, colitis, and irritable bowel syndrome also have been recorded (Menéndez Fernández-Miranda et al., 2025; Veraldi et al., 2022). *D. fragilis* infection rates vary from 0.04 % to over 86 %, depending on the population studied, region, and diagnostic procedures employed (Church et al., 2010). Studies have indicated higher infection rates in humans from low-income countries and areas with inadequate sanitation. Molecular analysis of the small subunit (*SSU*) rRNA gene has identified two *D. fragilis* genotypes: genotype 1 and genotype 2. Most human and animal isolates belong to genotype 1, while only a few samples have been identified as genotype 2 (Ercan et al., 2024). To date, limited information on *D. fragilis* infection rates and genetic characteristics have been reported in Xinjiang Uygur Autonomous Region (hereinafter Xinjiang), China. This study aimed to investigate the *D. fragilis* infection status in children in southern Xinjiang and provide baseline data on the molecular characteristics of this protozoan.

## 2 Materials and methods

### 2.1 Ethics approval and consent to participate

This study was conducted in accordance with the “Ethical Review of Biomedical Research Involving Humans” issued by the National Health Commission, China. The research protocol involving human participants was approved by the Ethics Review Committee of Tarim University (Approval No. IRC-TARU-20170414-10). Parents or guardians of children were informed in writing about the study's purpose and procedures, and those consenting to their children's participation signed informed consent forms.

### 2.2 Fecal sample collection

**Fig. 1:**
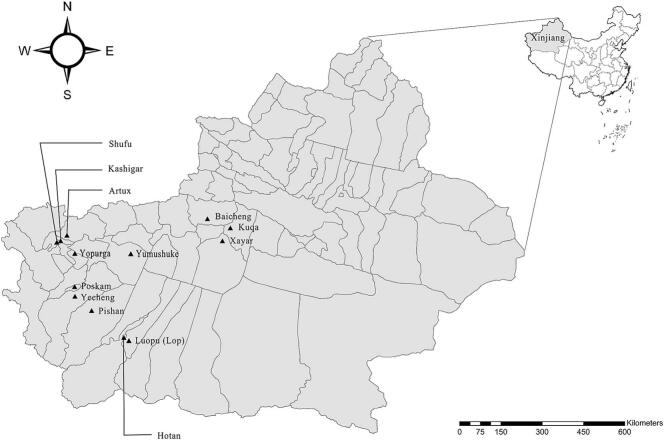
Geographic map showing the Xinjiang region of China and the 11 counties where samples were collected. The figure was originally designed by the authors under the software ArcGIS 10.2. The original vector diagram imported in ArcGIS was adapted from Natural Earth (http://www.naturalearthdata.com).

A total of 609 non-duplicate fecal sample were collected randomly from children (2 to 6 years old) attending kindergartens in 11 counties in Xinjiang, China, between February 2017 and January 2019 (Fig. 1). The cohort included 299 boys and 310 girls. Each sample was placed in a separated stool collection tube and transported to the laboratory at 4 °C. None of the children exhibited diarrhea or any other clinical symptoms at the time of sampling.

### 2.3 DNA extraction and PCR amplification

Genomic DNA was extracted from approximately 200 mg of each sample using the E.Z.N.A. Stool DNA Kit (Omega Bio-tek Inc., Norcross, GA, USA) and stored at −20 °C. PCR amplification targeting the *SSU* rRNA gene of *D. fragilis* was performed using specific primers DF1 and DF4 as previously described (Peek et al., 2004), generating a target fragments of ∼662 bp. Negative controls (nuclease-free water) and positive controls (human-derived ST3) were included in the PCR amplification.

### 2.4 Sequencing and phylogenetic analysis

All positive amplicons were sent to Youkang Biotechnology Co., Ltd., (Xinjiang, China) for sequencing. Raw sequence data were edited using DNASTAR Lasergene EditSeq software (version 7.1.0; http://www.dnastar.com/). Processed sequences were aligned using ClustalX (version 2.1; http://www.clustal.org/) with reference sequences retrieved from GenBank. A phylogenetic tree was constructed in MEGA11 using the Neighbor-Joining (NJ) method based on the Kimura 2-parameter model to analyze the genetic relationships of *D. fragilis*. The robustness of clusters was assessed using 1000 replicates; only branch support values above 50 % were retained. Sequences generated in this study were deposited in the GenBank database under accession numbers PV186784 and PV186785.

### 2.5 Statistical analysis

All statistical analyses were performed with the software IBM SPSS Statistics (www.ibm.com/products/spssstatistics). Fisher's exact test was used to study the difference of the infection rates. A two-sided *p*-value <0.05 was deemed significant.

## 3 Results and discussion

Of the 609 samples tested, 27 were *D. fragilis*-positive, yielding an infection rate of 4.4 % (27/609) (Table 1). This rate is higher than those reported in children in Pakistan (1.3 %, 2/150) (Bukhari et al., 2023), Gabon (4.0 %, 4/100) (Oyegue-Liabagui et al., 2020), Portugal (2.8 %, 4/144) (Júlio et al., 2015) and Vietnam (4.2 %, 2/48) (Ögren et al., 2016). However, it is lower than rates in children in Turkey (8.0 %, 4/50) (Özkan-Ahmetoğlu et al., 2023), Brazil (10.3 %, 16/156) (Oliveira-Arbex et al., 2021), Netherlands (15.3 %, 9/59) (Holtman et al., 2017), Spain (29.8 %, 17/57) (Montraveta-Querol et al., 2022), and Sweden (68.63 %, 70/102) (Ögren et al., 2015). Detection rates may vary depending on the methods used. Infection rates in China are likely underestimated due to the limited number of PCR-based studies on *D. fragilis*.

**Table 1:** Infection rate and genotype of *D. fragilis* in children in Southern Xinjiang.

Group	No. positives/ No. samples	Prevalence	95 % CI (%)	*p*-value	Genotype (n)
Sampling site					
Yopurga	10/63	15.9 %	6.1–25.7	–	Genotype 1 (10)
Yecheng	8/89	9.0 %	2.5–15.5	0.212	Genotype 1 (8)
Hotan	4/80	5.0 %	0–10.4	0.045	Genotype 1 (4)
Shufu	2/48	4.2 %	0–10.9	0.065	Genotype 1 (2)
Poskam	1/35	2.9 %	0–9.8	<0.001	Genotype 1 (1)
Pishan	1/37	2.7 %	0–9.3	<0.001	Genotype 1 (1)
Lop	1/109	0.9 %	0–3.2	<0.001	Genotype 1 (1)
Tumushuke	0/62	0	–	–	–
Payzawat	0/25	0	–	–	–
Kuqa	0/38	0	–	–	–
Baicheng	0/23	0	–	–	–
Tota	27/609	4.4 %	2.7–6.2	<0.001	Genotype 1 (27)
Age (months)					
0–23	2/62	3.2 %	0–8.4	0.883	Genotype 1 (12)
24–47	11/235	4.7 %	1.8–7.6	0.999	Genotype 1 (11)
48–72	14/312	4.5 %	2.0–6.9	0.999	Genotype 1 (14)
Gender					
Female	14/310	4.5 %	2.0–6.9	–	Genotype 1 (14)
Male	13/299	4.4 %	1.9–6.8	0.999	Genotype 1 (13)

Infection rates varied significantly among the seven positive counties, ranging from 0.9 % to 15.9 %. The highest infection rate was in Yopurga (15.9 %, 10/63), and the lowest was in Lop (0.9 %, 1/109). No *D. fragilis* infection was detected in children from the counties of Tumushuke, Payzawat, Baicheng, or Kuqa. The infection rate was 4.4 % (13/299) in boys and 4.5 % (14/310) in girls; no significant difference (*p* > 0.05) was observed between genders. Similar non-significant findings have been observed in children in Turkey (Özkan-Ahmetoğlu et al., 2023), Italy (Calderaro et al., 2014), and Portugal (Júlio et al., 2015), suggesting no significant association between the occurrence of *D. fragilis* and gender.

Although the role of *D. fragilis* in causing gastrointestinal pathology remains controversial, multiple studies describe clinical symptoms attributable to its infection (Menéndez Fernández-Miranda et al., 2025), with symptom duration ranging from days to two years. All children in this study were asymptomatic at the time of sampling. Without an appropriate control group, we cannot definitively confirm the pathogenicity of the *D. fragilis* strains detected in children in Xinjiang, China.

**Fig. 2:**
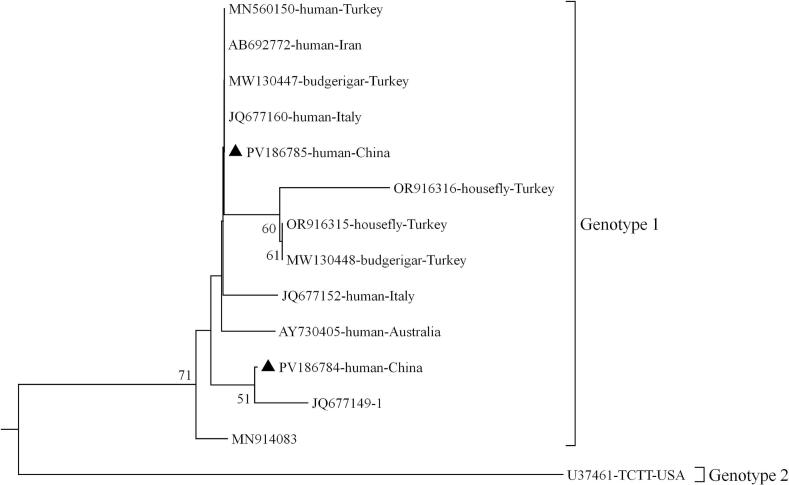
Phylogenetic relationships among representative sequences of the *Dientamoeba fragilis* small subunit ribosomal RNA (*SSU* rRNA) genes obtained from China, using the neighbor-joining method. Bootstrap values greater than 50 % from 1000 pseudo replicates are shown. Isolates identified in this study are indicated by filled triangles.

Sequencing of the 27 positive amplicons revealed that all *D. fragilis* isolates belonged to genotype 1. Two sequence types were identified (designated genotype 1a and genotype 1b) differing by only one single nucleotide polymorphism (SNP) at position 189 of the *SSU* rRNA gene (C/T substitution). Genotype 1a showed 100 % homology to JQ677149 (derived from human in the UK), and genotype 1b were showed 100 % homology to JQ677147 (derived from human in the UK) (Fig. 2). To date, two *D. fragilis* genotypes have been identified. Genotype 1 predominates in both symptomatic and asymptomatic populations across different age groups, while genotype 2 has been rarely documented and has primarily been isolated from pigs (Menéndez Fernández-Miranda et al., 2025). Notably, genotype 2 was recently reported in children from low-income neighborhoods in São Paulo, Brazil (Oliveira-Arbex et al., 2021). The factors influencing the genetic diversity of *D. fragilis* remain unclear.

Genotype 1 of *D. fragilis* has also been detected as the dominant or sole genotype in various animals, including pigs, budgerigars, and cattle (Cacciò et al., 2012; Yetismis et al., 2022; Yildiz and Erdem Aynur, 2022). Consequently, the zoonotic potential of this parasite has become a topic of increasing research interest. A recent study identified *D. fragilis* in houseflies, with all positive isolates assigned to genotype 1 based on phylogenetic analysis (Ercan et al., 2024). A growing consensus suggests possible cross-species transmission of *D. fragilis*, particularly since a cyst stage has been described (Munasinghe et al., 2013) and the fecal-oral route is recognized as the primary transmission route. Therefore, the genotype 1 *D. fragilis* detected in children may originate from livestock, pets, or houseflies. Because no direct evidence was found in this study, this hypothesis is speculative and requires confirmation through future studies involving animal and environmental samples.

## 4 Conclusions

The data presented here provide insights into the prevalence and genetic diversity of *D. fragilis* infections in asymptomatic young children in Xinjiang, China. Our findings confirm the presence of *D. fragilis* in this population was not associated with clinical symptoms. The genetic homogeneity observed suggests a clonal distribution pattern.

## CRediT authorship contribution statement

**Yafei Zhao:** Writing – original draft, Validation, Investigation, Formal analysis, Data curation. **Wenxuan Ma:** Writing – review & editing, Writing – original draft, Methodology, Conceptualization. **Duoduo Su:** Writing – review & editing, Supervision, Investigation, Data curation. **Zhenjie Zhang:** Writing – review & editing, Validation, Supervision, Software. **Aiyun Zhao:** Methodology, Investigation, Formal analysis, Data curation, Conceptualization. **Fuchang Yu:** Writing – review & editing, Supervision, Project administration, Investigation, Data curation, Conceptualization. **Meng Qi:** Writing – review & editing, Supervision, Project administration, Investigation, Funding acquisition, Data curation, Conceptualization.

## Declaration of competing interest

The authors declare that they have no competing interests.

## Data Availability

Representative nucleotide sequences generated in this study were deposited in the GenBank database under the Accession Numbers PV186784 and PV186785.
